# 4317 Using Failure Modes and Effects Analysis to Guide Adaptation of an Evidence-Based Parenting Program for Mothers with Substance Use Disorders

**DOI:** 10.1017/cts.2020.445

**Published:** 2020-07-29

**Authors:** Elizabeth Peacock-Chambers, Peter Friedmann, Nancy Byatt, Nancy Suchman, Emily Feinberg

**Affiliations:** 1 Tufts University; 2 UMMS-Baystate; 3 UMMS; 4 Yale School of Medicine; 5 Boston Medical Center

## Abstract

OBJECTIVES/GOALS: To identify possible failures that could occur in the delivery of an evidence-based parenting program for mothers with substance use disorders (SUD) through existing home-visiting services, and to develop solutions to the most significant failures. METHODS/STUDY POPULATION: Using failure modes and effects analysis (FMEA) methodology, we conducted two 2-hour advisory panel discussions with 15 people from a variety of disciplines and life experiences related to SUDs. The intervention delivery process included five steps: (1) Recruitment, (2) Screening, (3) Matching, (4) Enrollment in person, and (5) Intervention delivery. Participants collectively determined possible failures, causes, and consequences. Participants then agreed on three scores (Likert Scale 0-10) for the likelihood of occurrence, detection, and severity of the failure, with 10 being the highest likelihood, difficulty detecting, or severity. A risk priority number (RPN) was calculated as the product of the 3 scores (maximum RPN = 1,000). The group then identified possible solutions for failures with higher RPNs. RESULTS/ANTICIPATED RESULTS: For each step in the process we identified the following number of failure nodes and RPN scores: (1) recruitment: 13 failures; RPN = 800, (2) screening: 102 failures; RPN = 10, (3) matching: 4 failures: RPN = 490, (4) enrollment: 6 failures; RPN = 80, (5) delivery: 11 failures; RPN = 80. The most critical failures related to recruitment and were perceived as being caused by potential development of mistrust in the community. Participants strongly encouraged the use of “strengths-based language,” clear referral plans for mothers that did not qualify, and inclusion of mothers that did not have custody of their children. These findings resulted in changes to the screening script, enrollment procedures, and inclusion criterial for the program. DISCUSSION/SIGNIFICANCE OF IMPACT: FMEA methodology was particularly effective in identifying possible failures for the integration of an evidence-based parenting program into existing home-visiting services as they related to the psychological safety of mothers with SUDs. The process resulted in direct changes to procedures for the anticipated program integration and study.

